# Synergistic Effect of QNZ, an Inhibitor of NF-κB Signaling, and Bone Morphogenetic Protein 2 on Osteogenic Differentiation in Mesenchymal Stem Cells through Fibroblast-Induced Yes-Associated Protein Activation

**DOI:** 10.3390/ijms24097707

**Published:** 2023-04-22

**Authors:** Fei Huang, Hai Wang, Ying Zhang, Guozhen Wei, Yun Xie, Gui Wu

**Affiliations:** 1 Department of Orthopedics, The First Affiliated Hospital, Fujian Medical University, Fuzhou 350005, China; feifeigood2148@126.com (F.H.);; 2 Central Laboratory, The First Affiliated Hospital, Fujian Medical University, Fuzhou 350005, China; 3 Department of Orthopedics, National Regional Medical Center, Binhai Campus of the First Affiliated Hospital, Fujian Medical University, Fuzhou 350212, China

**Keywords:** QNZ, BMP2, osteoblast differentiation, MSCs, fibroblasts

## Abstract

Biomaterials carrying recombinant human bone morphogenetic protein 2 (BMP2) have been developed to enhance bone regeneration in the treatment of bone defects. However, various reports have shown that in the bone repair microenvironment, fibroblasts can inhibit BMP2-induced osteogenic differentiation in mesenchymal stem cells (MSCs). Thus, factors that can target fibroblasts and improve BMP2-mediated osteogenesis should be explored. In this project, we focused on whether or not an inhibitor of the NF-κB signaling pathway, QNZ (EVP4593), could play a synergistic role with BMP2 in osteogenesis by regulating the activity of fibroblasts. The roles of QNZ in regulating the proliferation and migration of fibroblasts were examined. In addition, the effect of QNZ combined with BMP2 on the osteogenic differentiation of MSCs was evaluated both in vitro and in vivo. Furthermore, the detailed mechanisms by which QNZ improved BMP2-mediated osteogenesis through the modulation of fibroblasts were analyzed and revealed. Interestingly, we found that QNZ inhibited the proliferation and migration of fibroblasts. Thus, QNZ could relieve the inhibitory effects of fibroblasts on the homing and osteogenic differentiation of mesenchymal stem cells. Furthermore, biomaterials carrying both QNZ and BMP2 showed better osteoinductivity than did those carrying BMP2 alone both in vitro and in vivo. It was found that the mechanism of QNZ involved reactivating YAP activity in mesenchymal stem cells, which was inhibited by fibroblasts. Taken together, our results suggest that QNZ may be a candidate factor for assisting BMP2 in inducing osteogenesis. The combined application of QNZ and BMP2 in biomaterials may be promising for the treatment of bone defects in the future.

## 1. Introduction

Bone has strong self-healing abilities. Thus, bone repair can be initiated to regenerate bone tissue when defects or fractures occur [1]. However, large or complex bone defects frequently exceed the self-healing ability of bones, leading to bone non-union [2,3]. Bone non-union is a chronic medical condition that not only affects the function of bones but can also impact the affected individual’s psychological and economic well-being [4]. Therefore, it is important to improve bone repair in patients with large bone defects. Usually, the self-healing ability of bone is not enough to repair the defects that occur as a result of bone non-union. Thus, medical interventions, such as bone grafting or bone tissue engineering, must be considered [5,6]. In addition, the application of the appropriate growth factors or bone matrix plays an important role in the treatment of bone non-union [7].

Bone morphogenetic protein 2 (BMP2), a member of the transforming growth factor-β (TGF-β) superfamily, plays a key role in osteogenesis during embryonic development and the repair of bone defects [8]. BMP2 can induce the osteogenic differentiation of mesenchymal stem cells (MSCs) during bone repair. The homing and osteogenic differentiation of MSCs are important factors in healing bone defects [9,10,11]. Recombinant human BMP2 has been developed to enhance the growth of bone tissue in a clinical setting. However, the half-life of BMP2 is short and the risk of local inflammatory response limits the efficacy of BMP2 in the treatment of bone defects [12,13,14]. As osteogenesis is a process that involves the participation of multiple signaling pathways and diverse types of cytokines [1,15], the application of BMP2 combined with other cytokines has been explored to enhance bone regeneration. For example, biomaterials carrying both VEGF and BMP2 have been used to improve osteogenesis in vivo [16,17].

Usually, bone repair under physiological conditions has two fates: bone union or scar union. It seems that there is competition between bone regeneration and scar formation in the bone defect region. Fibroblasts, as the main effector cells that can induce the formation of scar tissue, have been reported to inhibit the differentiation of MSCs into osteoblasts via various pathways. Fibroblasts can secrete Gremlin1, which inhibits BMP-mediated osteoblast differentiation [18]. Human gingival fibroblasts can suppress osteogenesis in MSCs by regulating the expression of miR-101-3p and ETS variant transcription factor 1 (ETV1) [19]. In addition, BMP2 can enhance the formation of fibrous scars during bone repair, which can inhibit osteogenesis [18,19,20]. Thus, targeting fibroblasts may be a potential method to improve the efficacy of BMP2-induced osteogenesis.

In this project, we found that QNZ, an inhibitor of the NF-κB signaling pathway [21], enhanced BMP2-mediated osteogenesis. QNZ inhibited the proliferation and migration of fibroblasts while promoting apoptosis. Co-treatment with QNZ and BMP2 was found to relieve the inhibitory effects of fibroblasts on the homing and osteogenic differentiation of MSCs. The localization and activity of Tafazzin/Yes-associated protein (TAZ/YAP) have previously been shown to affect the cell fate of MSCs. The nuclear localization of TAZ/YAP drives osteogenesis in MSCs, while the localization of TAZ/YAP to the cytoplasm facilitates adipogenesis [22]. In this research, QNZ was found to promote the nuclear localization of YAP in MSCs. On the other hand, the localization of YAP to the cytoplasm was induced by fibroblasts under co-culture conditions. Furthermore, biomaterials carrying both BMP2 and QNZ were shown to achieve better osteogenic efficacy both in vivo and in vitro than those carrying BMP2 or QNZ alone. Taken together, our results show that QNZ may be a new candidate factor for combination with BMP2 to promote osteogenesis by targeting fibroblasts. 

## 2. Results

### 2.1. QNZ Inhibited the Proliferation and Migration of Fibroblasts

**Figure 1:**
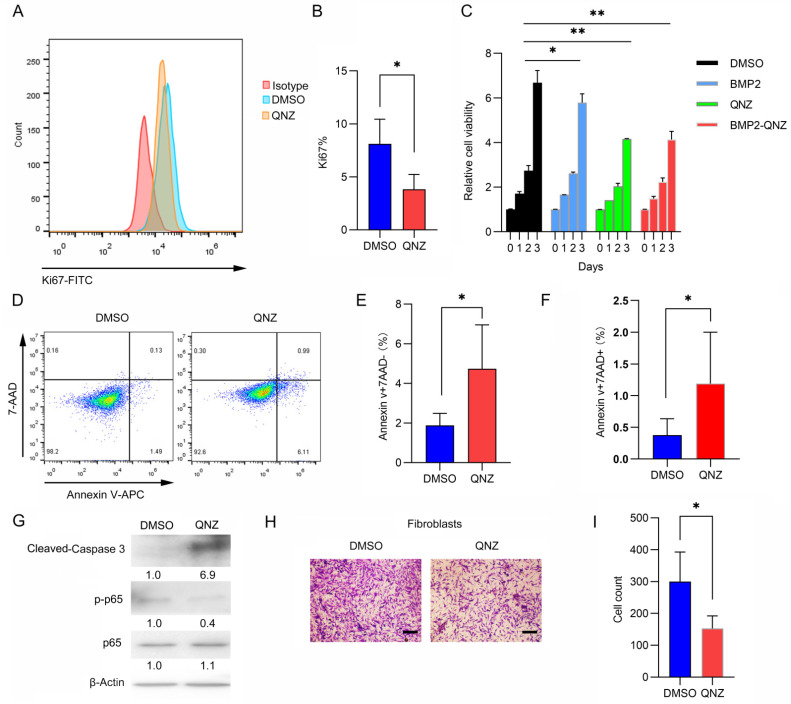
QNZ, an inhibitor of NF-κB signaling, inhibited the proliferation and migration of fibroblasts. (**A**) Representative flow cytometry image of fibroblasts. Fibroblasts were treated with control vehicle or 10 μM QNZ for 48 h before cell samples were collected and subjected to Ki-67-FITC staining. (**B**) Quantitative analyses of Ki67-positive fibroblasts in (**A**). At least three experiments were conducted and analyzed (*n* = 3). * *p* < 0.05, ** *p* < 0.01. (**C**) About 3000 fibroblasts were cultured in each well of 96-well plates with control vehicle or 10 μM QNZ for the indicated time. Relative cell viability (the OD values at 450 nm for each group normalized to the vehicle-treated group) was measured with a cell-counting kit. At least three experiments were conducted and analyzed (*n* = 3). * *p* < 0.05, ** *p* < 0.01. (**D**) Representative flow cytometry image of fibroblasts. Fibroblasts were treated with control vehicle or 10 μM QNZ for 48 h and cell samples were collected and subjected to Annexin V and 7-AAD staining. (**E**) Quantitative analyses of Annexin V-positive and 7-AAD-negative fibroblasts in (**D**). At least three experiments were conducted and analyzed (*n* = 3). * *p* < 0.05. (**F**) Quantitative analyses of Annexin V-positive and 7-AAD-positive fibroblasts in (**D**). At least three experiments were conducted and analyzed (*n* = 3). * *p* < 0.05. (**G**) Fibroblasts were treated with control vehicle or 10 μM QNZ for 48 h and cell samples were collected to subjected to Western blotting. The proteins indicated were examined. The original gels can be seen in the Supplementary Materials. The band intensity of each protein was analyzed and normalized to the band intensity of β-Actin in each group. Then, the relative band intensity of the QNZ group was normalized to the vehicle group, as shown below the band. (**H**) Fibroblasts were cultured in Transwell plates with control vehicle or 10 μM QNZ for 24 h. The cells that invaded the bottom were stained with crystal violet (representative images shown). Scale bar—100 μm. (**I**) Average cell numbers of at least three fields in (**H**) (*n* = 3). * *p* < 0.05.

We first examined whether QNZ could inhibit the proliferation and migration of fibroblasts. We found that the QNZ treatment inhibited the expression of Ki67, a proliferation marker, in fibroblasts (Figure 1A,B). Moreover, QNZ significantly suppressed the proliferation of fibroblasts within 3 days, and BMP2 treatment did not reverse this process (Figure 1C). Furthermore, QNZ induced apoptosis in fibroblasts. As shown in Figure 1D–F, both the percentages of AnnexinV^+^7AAD^-^ fibroblasts and AnnexinV^+^7AAD^+^ fibroblasts increased after treatment with QNZ, indicating that QNZ enhanced both early and late apoptosis in fibroblasts. Cleaved Caspase 3, a marker of apoptosis, was also present in fibroblasts when the activity of the NF-κB signaling pathway was inhibited by QNZ (Figure 1G). In addition, fibroblast migration was also attenuated by the QNZ treatment (Figure 1H,I). These results indicate that QNZ can inhibit the proliferation and migration of fibroblasts, which could make it a candidate factor to improve osteogenesis by inhibiting the functions of fibroblasts.

### 2.2. QNZ Promoted BMP2-Mediated Homing and Osteoblast Differentiation in MSCs When Co-Cultured with Fibroblasts

**Figure 2:**
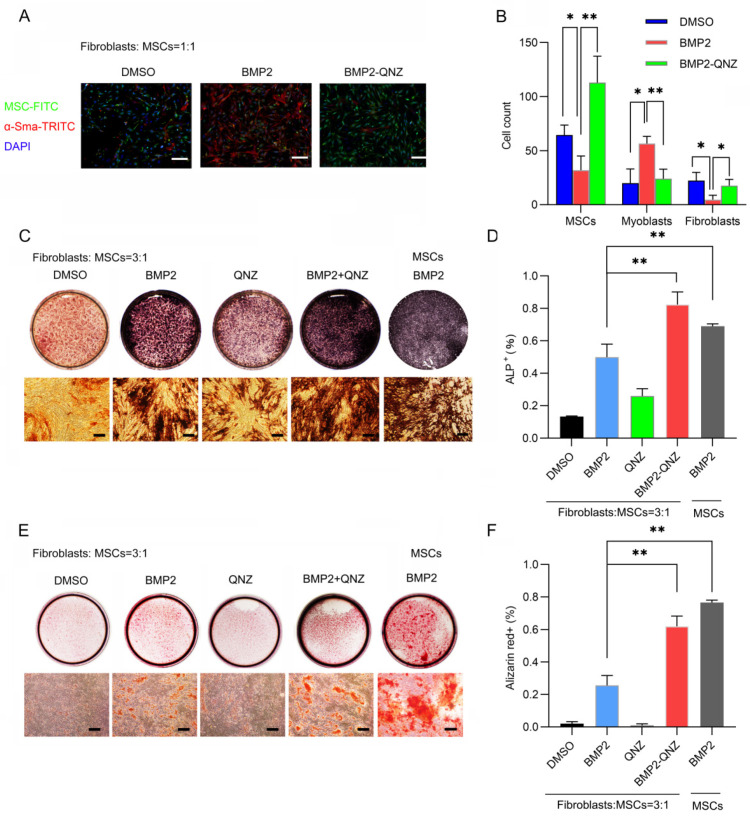
QNZ, an inhibitor of NF-κB signaling, reversing inhibition of BMP2-mediated homing and osteogenic differentiation in MSCs by fibroblasts. (**A**) Representative immunofluorescence staining images of fibroblasts and MSCs that migrated through the Transwell membrane after they were co-cultured and treated with control vehicle, 200 ng/mL of BMP2 or 10 μM QNZ. Green cells are MSCs, red cells are α-SMA^+^ cells (myofibroblasts) and blue represents DAPI, a marker of the cell nuclei. Scale bar—100 μm. (**B**) Quantitative analysis of number of MSCs, myofibroblasts with high a-SMA intensity and fibroblasts with low a-SMA intensity in (**A**). Average cell numbers of at least three fields in (**A**) are shown (*n* = 3); * *p* < 0.05 and ** *p* < 0.01. (**C**) ALP staining of MSCs after co-culturing with fibroblasts and treatment with control vehicle, 200 ng/mL of BMP2 or 10 μM QNZ for 7 days. Scale bar—100 μm. (**D**) Quantitative analysis of percentage of ALP^+^ cells in (**C**). At least three experiments were conducted and analyzed (*n* = 3). ** *p* < 0.01. (**E**) Alizarin red staining of calcium after MSCs were co-cultured with fibroblasts and treated with control vehicle, 200 ng/mL of BMP2 or 10 μM QNZ for 14 days. Scale bars—100 μm. (**F**) Quantitative analysis of percentage of Alizarin red^+^ areas in (**E**). At least three experiments were conducted and analyzed (*n* = 3). ** *p* < 0.01.

We next investigated how QNZ affected BMP2-mediated migration and osteoblast differentiation in MSCs when they were co-cultured with fibroblasts. Fibroblasts and MSCs were co-cultured on the upper insert of Transwell with 8 μm pores. As shown in Figure 2A,B, the migration of myofibroblasts was enhanced while the migration of MSCs was inhibited when BMP2 was added. However, the QNZ treatment was found to enhance the migration of MSCs while inhibiting the migration of myofibroblasts. These results indicate that QNZ can rescue the migration of MSCs inhibited by myofibroblasts. When bone defects caused by injuries exceed the self-repair ability of bone, fibrous tissues form scars to connect the defective bones [23]. Fibroblasts co-exist with MSCs in the bone repair microenvironment. Thus, we examined the effect of QNZ on the differentiation of MSCs into osteoblasts in a system in which fibroblasts and MSCs were co-cultured. We found that the fibroblasts could inhibit BMP2-mediated osteogenic differentiation in the MSCs. However, QNZ was shown rescue BMP2-mediated osteogenic differentiation the in MSCs when they were co-cultured with fibroblasts. At the early stage of osteoblast differentiation, the percentage of ALP^+^ cells decreased when the MSCs were co-cultured with fibroblasts in contrast to when the MSCs were cultured alone. Interestingly, the percentage of ALP^+^ cells increased significantly in the QNZ-BMP2-treated group compared to the BMP2-treated group when the MSCs and fibroblasts were co-cultured (Figure 2C). Moreover, more calcium nodules (stained by Alizarin red), which indicate the formation of mature osteoblasts, were observed in the QNZ-BMP2-treated group than in the BMP2-treated group, suggesting that QNZ also rescued BMP2-mediated osteogenesis inhibited by fibroblasts in the late stage (Figure 2D). In addition, the QNZ-BMP2 treatment did not affect the proliferation of MSCs (Supplementary Figure S1A). Furthermore, the expression of markers of differentiated bone—*Runx2*, *Ocn* and *Osx*—also verified that the treatment with QNZ-BMP2 enhanced osteogenesis more significantly than the treatment with BMP2 alone when the fibroblasts and MSCs were co-cultured (Supplementary Figure S1B). Taken together, these results suggest that QNZ can improve BMP2-mediated osteogenesis by inhibiting the activity of fibroblasts in vitro.

### 2.3. QNZ Treatment Promoted an Osteogenic Phenotype in MSCs When Co-Cultured with Fibroblasts

**Figure 3:**
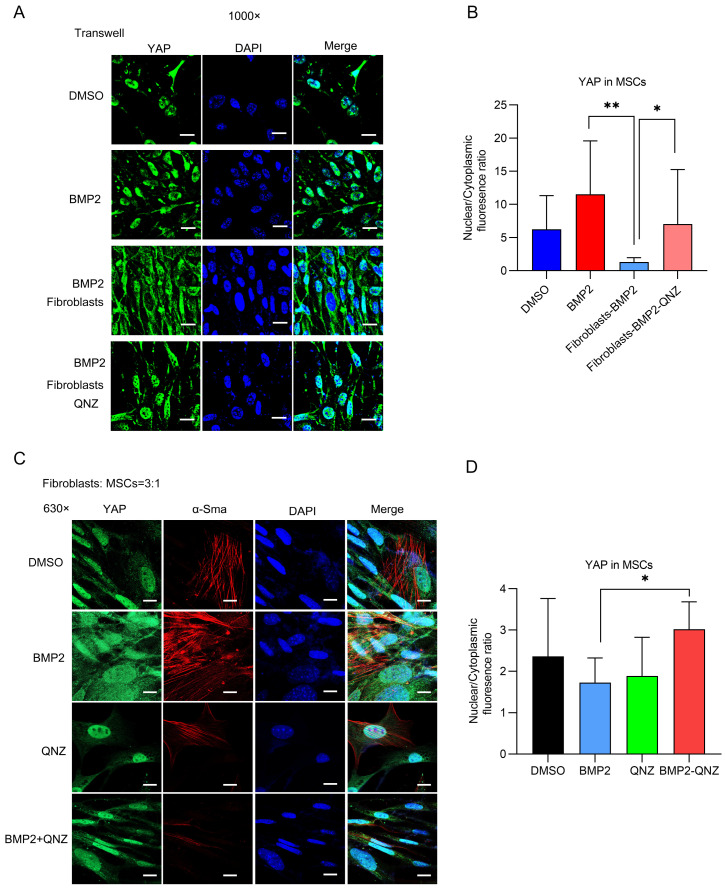
QNZ, an inhibitor of NF-κB signaling, recovered the activity of YAP in MSCs inhibited by fibroblasts. (**A**) Representative immunofluorescence staining images of MSCs after co-culturing with fibroblasts in Transwell plates and treatment with control vehicle, 200 ng/mL of BMP2 or 10 μM QNZ for 24 h. Green represents YAP while blue indicates DAPI. Scale bar—100 μm. (**B**) Quantitative analysis of the ratio of the nuclear fluorescence intensity to cytoplasmic fluorescence intensity of YAP in MSCs in (**A**). At least three experiments were conducted and analyzed (*n* = 3). * *p* < 0.05 and ** *p* < 0.01. (**C**) Representative immunofluorescence staining images of cells derived from MSCs directly co-cultured with fibroblasts and treated with control vehicle, 200 ng/mL of BMP2 or 10 μM QNZ for 24 h. Green represents YAP, red represents α-SMA and blue indicates DAPI. Scale bar—100 μm. (**D**) Quantitative analysis of ratio of nuclear fluorescence intensity to cytoplasmic intensity of YAP in the MSCs in (**C**). At least three experiments were conducted and analyzed (*n* = 3). * *p* < 0.05.

As fibroblasts co-exist with MSCs in the bone repair microenvironment, they may create competition for space and nutrients, which could affect the state of YAP activity in MSCs. In MSCs, the nuclear localization of TAZ/YAP drives osteogenesis, while the localization of TAZ/YAP to the cytoplasm facilitates adipogenesis. We further analyzed whether or not the localization of YAP in MSCs was affected by fibroblasts and whether or not QNZ played a role in this process. We found that fibroblasts could induce YAP to translocate to the cytoplasm from the nucleus in MSCs under both direct and indirect co-culture conditions (Figure 3A–D). QNZ was found to promote the relocalization of YAP to the nucleus, even when MSCs were co-cultured with fibroblasts. These results indicate that fibroblasts can induce MSCs to display a phenotype that is prone to adipogenesis when the two are co-cultured, while QNZ can inhibit this effect and rescue the nuclear localization of YAP, facilitating the osteogenic differentiation of MSCs.

### 2.4. BMP2/QNZ/Collagen I Biomaterials Enhanced the Osteogenic Differentiation of MSCs

**Figure 4:**
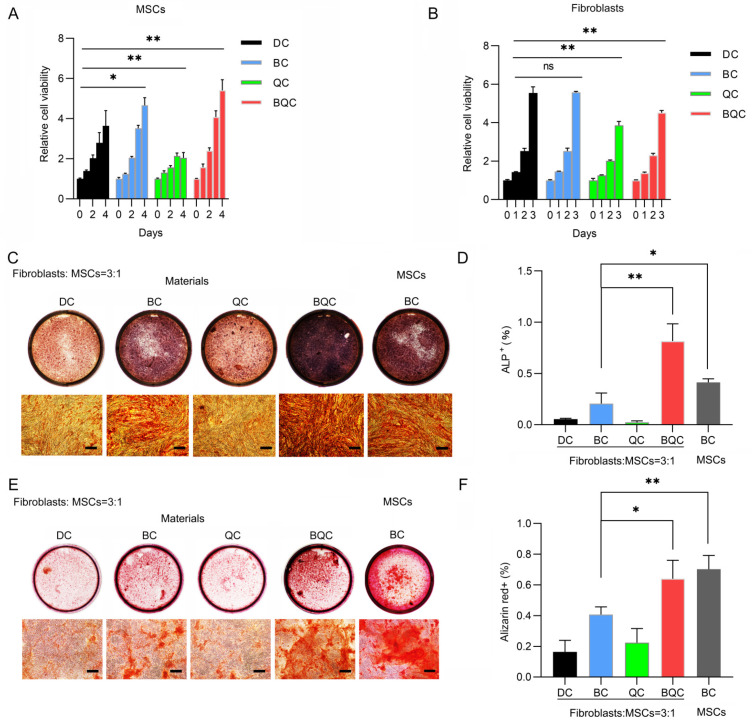
BMP2/QNZ/Collagen I biomaterials enhanced osteogenic differentiation of MSCs. (**A**) About 3000 MSCs cultured in each well of 96-well plates with DMSO/Collagen I (DC), BMP2/Collagen I (BC), QNZ/Collagen I (QC) or BMP2/QNZ/Collagen I (BQC) for the indicated time. Relative cell viability (OD values at 450 nm of each group normalized to DC group) was measured with a cell-counting kit. At least three experiments were conducted and analyzed (*n* = 3). * *p* < 0.05 and ** *p* < 0.01. (**B**) About 3000 fibroblasts cultured in each well of 96-well plates with DC, BC, QC and BQC. Relative cell viability (OD values at 450 nm of each group normalized to DC group) was measured with a cell-counting kit. At least three experiments were conducted and analyzed (*n* = 3). * *p* < 0.05 and ** *p* < 0.01. (**C**) ALP staining of MSCs after co-culturing with fibroblasts and treatment with DC, BC, QC or BQC for 7 days. Scale bar—100 μm. (**D**) Quantitative analysis of percentage of ALP^+^ cells in (**C**). At least three experiments were conducted and analyzed (*n* = 3). * *p* < 0.05 and ** *p* < 0.01. (**E**) Alizarin red staining of calcium after MSCs were co-cultured with fibroblasts and treated with DC, BC, QC or BQC for 14 days. Scale bar—100 μm. (**F**) Quantitative analysis of percentage of Alizarin red^+^ areas in (**E**). At least three experiments were conducted and analyzed (*n* = 3). * *p* < 0.05 and ** *p* < 0.01.

To study the osteogenic effects of the co-delivery of QNZ and BMP2 in vitro and in vivo, we synthesized biomaterials containing BMP2 and QNZ. Collagen I was utilized as the carrier, and four kinds of materials were synthesized: Collagen I/DMSO (DC), Collagen I/BMP2 (BC), Collagen I/QNZ (QC) and Collagen I/BMP2/QNZ (BQC). The materials were frozen and dried before undergoing characterization via SEM. As shown in Supplementary Figure S2A, many pores were formed in the materials. Consistent with the function of BMP2 and QNZ, BC and BQC showed no cytotoxicity to the MSCs (Figure 4A); however, BQC was found to significantly inhibit the proliferation of fibroblasts (Figure 4B). Moreover, BQC was shown to rescue osteogenic differentiation in the MSCs inhibited by fibroblasts in co-culture conditions (Figure 4C–F). Fibroblasts also inhibited BC-induced osteogenic differentiation in the MSCs. However, in the BQC group, both the percentage of ALP^+^ cells and the formation of calcium nodes increased more than in the BC group when the fibroblasts and MSCs were co-cultured. In addition, the expression of the osteogenic differentiation markers *Runx2*, *Ocn* and *Osx* also showed that the BQC treatment enhanced osteogenesis more significantly than the BC treatment did (Supplementary Figure S2B). These results indicate that the co-delivery of BMP2 and QNZ within Collagen I biomaterials can enhance BMP2-induced osteogenic differentiation in MSCs by targeting fibroblasts.

### 2.5. BMP2/QNZ/Collagen I Biomaterials Enhanced Osteogenesis In Vivo

**Figure 5:**
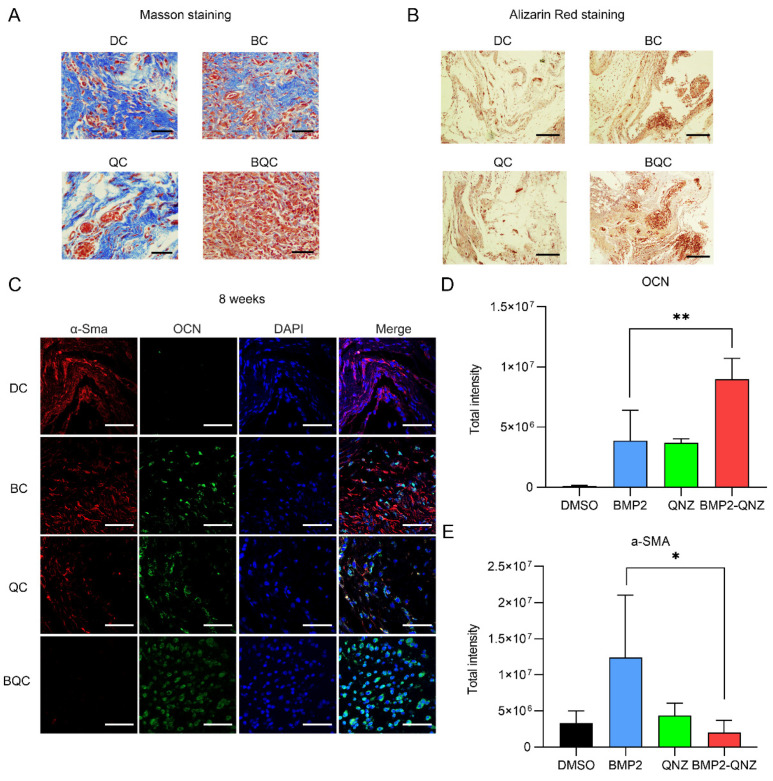
BMP2/QNZ/Collagen I biomaterials enhancing osteogenesis in vivo. (**A**) Masson staining of samples harvested from DMSO/Collagen I (DC)-, BMP2/Collagen I (BC)-, QNZ/Collagen I (QC)- and BMP2/QNZ/Collagen I (BQC)-induced ectopic bone formation experiments. Blue represents fibrous tissues and red indicates new bone. Scale bars of the 100× photos are 100 μm. (**B**) Alizarin red staining of samples harvested from DC-, BC-, QC- and BQC-induced ectopic bone formation experiments. Blue represents fibrous tissues and red indicates new bone. Scale bars of the 100× photos are 100 μm. (**C**) DC, BC, QC and BQC implanted in the muscle bag of mice for 8 weeks. Tissues were harvested for immunofluorescence analysis. Red—α-SMA; green—OCN; blue—DAPI. Scale bar—50 μm. (**D**) Each slice of the immunofluorescence staining image segmented into several small units for analysis. The scatter diagram illustrates the average total fluorescence intensity of OCN in at least three units (*n* = 5). ** *p* < 0.01. (**E**) Each slice of the immunofluorescence staining image segmented into several small units for analysis. The scatter diagram illustrates the average total fluorescence intensity of α-SMA in at least three units (*n* = 5). * *p* < 0.05.

**Figure 6:**
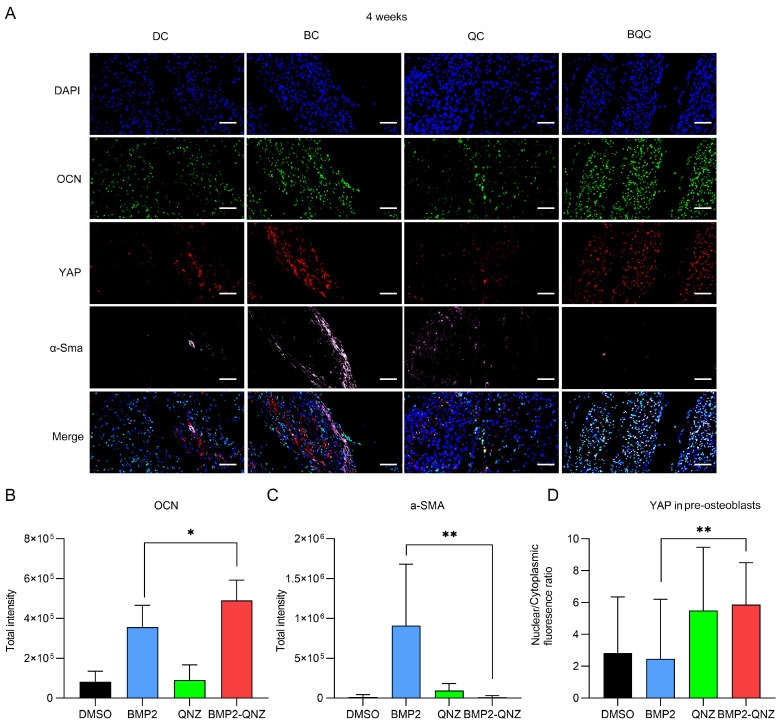
BMP2/QNZ/Collagen I biomaterials modulating YAP activity in MSCs via fibroblasts in vivo. (**A**) DMSO/Collagen I (DC), BMP2/Collagen I (BC), QNZ/Collagen I (QC) and BMP2/QNZ/Collagen I (BQC) implanted in the muscle bag of mice for 4 weeks. Tissues were harvested for immunofluorescence analysis. Red—YAP; pink—α-SMA, green—OCN; blue—DAPI. Scale bar—100 μm. (**B**) Each slice of the immunofluorescence staining image segmented into several small units for analysis. The scatter diagram illustrates the average total fluorescence intensity of OCN in at least three units (*n* = 5). * *p* < 0.05. (**C**) Each slice of the immunofluorescence staining image segmented into several small units for analysis. The scatter diagram illustrates the average total fluorescence intensity of α-SMA in at least three units (*n* = 5). ** *p* < 0.01. (**D**) Quantitative analysis of the ratio of nuclear fluorescence intensity to cytoplasmic fluorescence intensity of YAP in OCN-positive cells (*n* = 5). ** *p* < 0.01.

We further analyzed the functions of the BMP2/QNZ/Collagen I biomaterials in vivo. An ectopic osteogenesis model was created. DC, BC, QC and BQC were implanted into the muscle bags of the thigh in the C57BL/6 mice. After 8 weeks, the tissues were harvested and subjected to Masson and Alizarin red staining. As shown in Figure 5A, Masson staining revealed that BC could induce the formation of both fibrous scars (blue) and new bone tissue (red). However, BQC significantly reduced the formation of fibrous scars and enhanced the formation of new bone tissues. Alizarin red staining also showed that BQC could enhance the formation of new bone tissues more significantly than BC could (Figure 5B). The immunofluorescence staining showed that both α-Sma^+^ myofibroblasts and OCN^+^ osteoblasts existed in the BC-implanted tissues. However, the implantation of BQC was shown to reduce the number of α-Sma^+^ myofibroblasts and enhance the differentiation of OCN^+^ osteoblasts (Figure 5C–E). We further analyzed whether or not the BMP2/QNZ/Collagen I biomaterials could affect YAP activity in the OCN^+^ osteoblasts. Consistently, the nucleus/cytoplasm ratios of the OCN^+^ osteoblasts were higher in the BQC-implanted tissues than they were in the BC-implanted tissues, showing that BQC induced higher YAP activation than BC did (Figure 6A–D). These results are consistent with the experimental results obtained in vitro, indicating that BMP2/QNZ/Collagen I biomaterials can enhance osteogenesis via the inhibition of fibroblast activity in vivo. 

## 3. Discussion

The function of BMP2 in osteogenesis is significant and well-studied [24]. However, due to its short half-life and side effects in vivo, biomaterials that only carry BMP2 cannot achieve the desired effects in the treatment of most bone non-unions [12,13,14]. Thus, research has focused on improving the efficacy of BMP2 biomaterials for osteogenesis in recent years. Some researchers have attempted to optimize biomaterials that carry BMP2, while others have tried to add chaperones for BMP2 [3]. The surface characteristics of these materials and the therapeutic release strategies used for BMP2 are important for osteogenesis [3]. Previous reports have tried to expand the available areas for BMP2 and the cells to interact with each other [25,26,27]. Moreover, strategies focused on the controlled release of BMP2 can overcome the shortcomings caused by its short half-life [28,29,30]. Other studies have also tried to add other proteins to enhance osteogenesis in combination with BMP2, mainly growth factors such as FGFs, VEGFs and IGFs [31,32,33]. In this research, we reported a newly defined factor, QNZ, which can assist BMP2 in inducing osteogenesis. 

**Figure 7:**
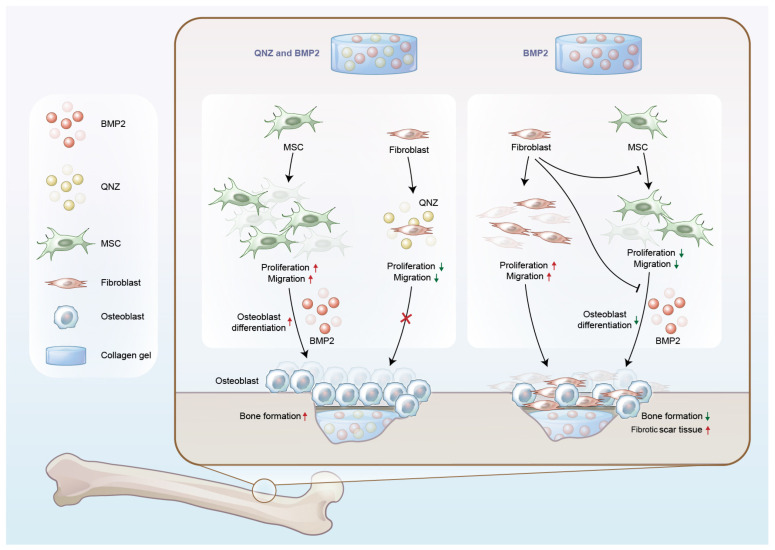
QNZ playing a synergistic role with BMP2 to induce osteogenesis by targeting fibroblasts. Fibroblasts can inhibit BMP2-mediated osteogenic differentiation in MSCs. Thus, the proliferation and migration of fibroblasts in the microenvironment of osteogenesis is harmful to bone repair, resulting in the formation of fibrous scars and the failure of bone formation. QNZ can inhibit the proliferation and migration of fibroblasts while also enhancing fibroblast apoptosis. Co-treatment with QNZ and BMP2 can reverse the inhibitory effects of fibroblasts on the homing and osteogenic differentiation of MSCs. Thus, QNZ may be a new candidate factor for combination with BMP2 to promote osteogenesis by targeting fibroblasts.

MSCs are the primary cells that participate in the regeneration of bones. MSCs can differentiate into diverse cell types, including adipocytes, osteoblasts, chondrocytes and (debatably) endothelial cells. The differentiation of MSCs into osteoblasts plays a key role in bone repair after injuries [34]. Thus, some studies have tried to engraft MSCs with BMP2-containing biomaterials to treat bone fractures [35,36,37]. However, the microenvironment of bone defects is complex and contains various types of cells, such as MSCs, fibroblasts and inflammatory cells. Thus, the functions of other types of cells in osteogenesis should be addressed in the treatment of bone defects. Biomaterials targeting macrophages and macrophage-derived osteoclasts have been developed to improve osteogenesis [11,38]. However, fibroblasts play an important role in the inhibition of osteoblast differentiation in MSCs. Few studies have focused on the development of materials that have effects on fibroblasts. Fibroblasts have been reported to inhibit osteogenesis through the secretion of BMP2 inhibitors or miRNAs [18,19,20]. Thus, we attempted to find an inhibitor to block the activity of fibroblasts and enhance the differentiation of MSCs into osteoblasts. The NF-κB signaling pathway has been reported to play a significant role in the activation of cancer-associated fibroblasts and the development of pathological fibrosis. NF-κB signaling activation promotes the proliferation and activation of fibroblasts by enhancing inflammation indirectly and has key effects on the proliferation and activation of fibroblasts directly [39,40]. QNZ is an inhibitor of the NF-κB signaling pathway [21]. Bone regeneration can be divided into two stages: endogenous stem cell homing to the sites of bone defects and the subsequent osteogenic differentiation of the recruited stem cells [41,42]. In this project, we found that QNZ could rescue BMP2-mediated migration and osteogenic differentiation in MSCs inhibited by fibroblasts. Moreover, the BMP2/QNZ/Collagen I biomaterials promoted osteogenesis in vitro and in vivo by targeting fibroblasts. Our results show that fibroblasts may also be a good target for the improvement of osteogenesis, in addition to MSCs and osteoclasts (Figure 7).

NF-κB signaling can regulate inflammation and the activity of fibroblasts [39,40,43]. In this project, we found that QNZ could inhibit the proliferation and migration of fibroblasts. QNZ also rescued BMP2-mediated osteogenic differentiation in MSCs inhibited by fibroblasts. The molecular mechanism underlying the effect of fibroblasts involves the translocation of YAP from the nucleus of the MSCs to the cytoplasm. YAP/TAZ can sense changes in the competition for space and nutrients between cells. When space and nutrients are rich, YAP/TAZ is active and stays in the nucleus. However, when space and nutrients are scarce and competition exists between cells, YAP/TAZ can be inactivated and translocated to the cytoplasm [44]. In addition, YAP/TAZ has been reported to play an important role in osteogenesis [22,45,46]. The nuclear localization of TAZ/YAP drives osteogenesis in MSCs, while the cytoplasmic localization of TAZ/YAP facilitates adipogenesis [22]. However, QNZ can help YAP translocate back to the nucleus even when MSCs are co-cultured with fibroblasts. The nuclear localization of YAP is beneficial to the osteogenic differentiation of MSCs. However, the mechanism underlying how QNZ functions during osteogenesis may be complex, because NF-κB signaling plays roles in various types of cells in the bone repair microenvironment. Moreover, the BMP2/QNZ/Collagen I biomaterials had more beneficial effects on osteogenesis than the BMP2/Collagen I biomaterials did in vivo. The effects of QNZ on inflammatory cells in the bone repair microenvironment in addition to the effects of fibroblasts and MSCs require further research.

In summary, we found that QNZ could relieve the inhibitory effects of fibroblasts on the homing and osteogenic differentiation of mesenchymal stem cells. QNZ was found to reactivate YAP activity in mesenchymal stem cells inhibited by fibroblasts. Furthermore, biomaterials carrying both QNZ and BMP2 showed improved osteo-inductivity. Thus, QNZ may be a good candidate factor for improving BMP2-mediated osteogenesis and can be used with BMP2 in bone substitute materials to enhance bone repair.

## 4. Materials and Methods

### 4.1. Antibodies and Reagents 

The following reagents were used in this study: BMP2 (R&D, 355-BM-100); QNZ (EVP4593, Selleck, S4902); Succinimide ester (CFSE, eBioscience, 65-0850-84); 4′,6-diamidino-2-phenylindole (DAPI, Solarbio, C0060); and Collagen I, Rat (ThermoFisher Scientific, A1048301). The antibodies used in this study were monoclonal anti-YAP (Cell Signaling Technology, 14074); anti-Ki67-fluorescein isothiocyanate isomer (anti-Ki67-FITC, BioLegend, 151211); anti-actin alpha 2, smooth muscle (anti-α-Sma, Servicebio, GB111364); anti-osteocalcin (anti-OCN, biorbyt, orb259644); Tetramethylrhodamine isothiocyanate (TRITC)-conjugated anti-rabbit antibody (abclonal, AS040) and FITC-conjugated anti-mouse antibody (abclonal, AS001). Tris-HCl, NaCl and other chemicals were purchased from Sigma.

### 4.2. Cell Culture

Mice mesenchymal stem cells were extracted from the bone marrow of C57BL/6 mice. All bone marrow cells were harvested from the tibias of C57BL/6 mice. Then, the bone marrow cells were cultured in a stem cell culture medium from Cyagen. Some cells attached to the plates while the other cells remained suspended. After 48 h, the suspended cells were discarded and the adherent cells were cultured for an additional two weeks to obtain primary mice bone marrow-derived mesenchymal stem cells (BMSCs). All the BMSCs used for the following experiments were from P2 to P8. Mouse embryonic fibroblast (MEF) cells were derived from C57BL/6 mice embryos. At day 13.5, embryos were harvested and fibroblasts were extracted from the back. The fibroblast cells were cultured in DMEM (Invitrogen) with 10% FBS (Hyclone). The number of passages of the MEF cells used for the subsequent experiments was less than five.

### 4.3. Alkaline Phosphatase (ALP) Staining

BMSCs from C57BL/6 mice were cultured in media for differentiation (α-MEM from Gibco with 10% fetal bovine serum (FBS), 50 μM ascorbic acid and 100 mM β-glycerophosphate) with the indicated treatment to induce osteogenic differentiation for seven days. The culture media were renewed at 1 d, 3 d and 5 d. ALP staining was performed using BCIP/NBT Alkaline Phosphatase Color Development Kit (Beyotime). Photos were taken using an Olympus IX50/70 microscope system.

### 4.4. Alizarin Red Staining

BMSCs from C57BL/6 mice were cultured in media for differentiation (α-MEM from Gibco with 10% FBS, 50 μM ascorbic acid and 100 mM β-glycerophosphate) with the indicated treatment to induce osteogenic differentiation for 14–21 days. The culture media were renewed at 1 d, 3 d, 5 d, 10 d, 15 d and 20 d. Afterwards, the cells were fixed with 95% ethanol for five minutes before staining with 1% Alizarin red (pH = 4.2) for 15 min. Photos were taken using an Olympus IX50/70 microscope system.

### 4.5. CFSE-Labeled MSCs

MSCs were cultured in 6-well plates to a confluency of 60~80%. Then, the MSCs were digested and resuspended in PBS at a concentration of 10^6^ cells/mL. The MSCs were treated with 2.5 μM CFSE and shaken in a 37 °C water bath for 10 min. Finally, the cells were washed with the complete culture medium twice before the experiments were carried out. 

### 4.6. Cell Proliferation

Aliquots of 3 × 10^3^ MSCs or MEFs were cultured in 96-well plates for the following treatment. After incubation periods of 1 d, 2 d, 3 d and 4 d, Cell Counting Kit-8 (Dongjindo Molecular Technologies, Inc., Tokyo, Japan) was used for calculating cell viability. Absorbance was measured at 450 nm using a Molecular Devices SpectraMax i3X system. For the next treatment, 1 × 10^5^ MEFs were cultured in 6-well plates. After 24 h of culture, the MEFs were harvested and subjected to Ki67-FITC staining. Flow cytometry was performed to examine the expression of Ki67 in MEFs using Accuri C6-BD. The results were analyzed using Flow J 10.4.

### 4.7. Apoptosis

Aliquots of 1 × 10^5^ MEFs were cultured in 6-well plates for the following treatment. After culturing for 24 h, the MEFs were harvested and subjected to Annexin V and 7-Aminoactinomycin D (7-AAD) staining (BioLegend, 640930). Flow cytometry was performed to examine apoptosis in the MEFs using Accuri C6-BD. The results were analyzed using Flow J 10.4.

### 4.8. Cell Migration

Aliquots of 5 × 10^3^ MSCs labeled with CFSE and 5 × 10^3^ MEFs (or 1 × 10^4^ MEFs alone) in serum-free DMEM culture media were cultured on the upper layer of Corning cell culture inserts with an 8.0 μm polycarbonate membrane. A DMEM culture medium with 10% FBS and the indicated cytokines or chemicals was added below the cell’s permeable membrane. Following an incubation period of 24 h at 37 °C and 5% CO_2_, the cells that had migrated through the membrane were harvested. For immunofluorescence, cells were fixed with 4% paraformaldehyde (Sigma) for 20 min. Then, 10% bovine albumin (BSA) was used to block the cells for 20 min at room temperature. Afterwards, the cells were incubated with anti-α-Sma (Servicebio, GB111364) overnight at 4 °C. On the second day, the cells were incubated with the TRITC-conjugated anti-rabbit antibody (Abclonal, AS040) for one hour at room temperature. DAPI (Solarbio, C0060) was used to stain the nucleus. Images were taken using a Zessis Axio Scope A1 FL for FISH microscope. For crystal violet staining, cells were fixed with methanol for 20 min and 0.1% crystal violet was added. Photos were taken using an Olymbus IX50/70 microscope system.

### 4.9. Synthesis of BMP2/QNZ Collagen Materials 

Aliquots of 3 mg/mL of a 150 μL Collagen I solution were mixed with 7.5 μg BMP2 or 20 μg QNZ. Then, the pH value of the Collagen I solution was adjusted to 7.4 with 10 M NaOH to crosslink the collagen fibers. The collagen materials were frozen, dried and stored at −20 °C for later use. The materials were synthesized and stored under aseptic conditions. Collagen materials with DMSO were labeled DMSO/Collagen I (DC); materials with BMP2 alone were named BMP2/Collagen I (BC); materials with QNZ alone were labeled QNZ/Collagen I (QC); and materials with both BMP2 and QNZ were named BMP2/QNZ/Collagen I (BQC). After the materials were synthesized, they were characterized using scanning electron microscopy (SEM, Verios G4). Each mouse was implanted with materials that contained 7.5 μg BMP2 or 20 μg QNZ.

### 4.10. Mice

The female C57BL/6 mice (6–8 weeks) used in this study were bred and maintained in a specific-pathogen-free animal facility at Fujian Medical University. The mice were euthanized via carbon dioxide asphyxiation. All animal experiments were approved by the Animal Ethical Committee of Fujian Medical University (FJMU IACUC 2021-0506). The C57BL/6 mice were anesthetized with an intravenous injection of 1.5% pentobarbital sodium (40 mg/kg body weight, Sigma, Las Vegas, NV, USA). A 1 cm skin incision was made along the thigh, and the myolemma was split longitudinally. The DC, BC, QC and BQC materials were implanted into the muscle, and the muscle and skin were sutured. Penicillin sodium (200,000 IU) was injected intramuscularly to prevent infection. The animals were sacrificed after 4 or 8 weeks, and the samples were harvested.

### 4.11. Masson Staining and Alizarin Red Staining

The tissue samples were embedded in paraffin, cut into 2.5 μm sections and dewaxed with xylene. Then, the sections were rehydrated with 100%, 95%, and 75% alcohol gradients. The sections were stained using a Masson staining kit (Solarbio, G1346) or Alizarin red staining kit (Solarbio, G1452). Finally, a neutral resin was used to mount the sections. Photos were taken using an Olympus BX53 microscope.

### 4.12. Immunofluorescence

For tissue sections, the above protocol for Masson staining was followed before incubation with primary antibodies. For cell samples, the cells were fixed with 4% paraformaldehyde (Sigma) for 20 min. Then, the cells were permeated with 0.2% Triton X-100 for 20 min at room temperature. BSA (10%) was used to block the cells for 20 min at room temperature. After, the cells were incubated with primary antibodies overnight at 4 °C. On the second day, the cells and sections were incubated with DAPI (Solarbio, C0060) and TRITC- or FITC-conjugated secondary antibodies for one hour at room temperature. Images were taken using a Zessis LSM 800 laser scanning confocal microscope.

### 4.13. Immunoblotting Assay

Immunoblotting assays were carried out as previously reported [47]. Cells were first lysed with a TNE buffer (10 mM Tris-HCl, 150 mM NaCl, 1 mM EDTA, and 0.5% NP40; pH = 7.5). Then, the cell lysates were mixed with a 4× loading buffer (40 mM Tris-HCl, 200 mM DTT, 4% SDS, 40% Glycerol, and 0.032% Bromophenol blue; pH = 8.0). The samples were run with 4% stacking gel and 10% separating gels. Then, the proteins on the gels were transferred to nitrocellulose filter membranes for incubation with antibodies. Membrane exposure was carried out using Thermo Pierce ECL and FluorChem E (Protein Simple). The original gels for Western blotting can be seen in the Supplementary Materials.

### 4.14. Quantitative Real-Time PCR

MSCs were cultured under different treatments for osteogenic differentiation. After 14 days, the total cell RNA of each sample was isolated using TRIzol (Invitrogen) and cDNA was synthesized with Revertra Ace (Promega, Madison, WI, USA). A real-time PCR was performed using an ABI QuantStudio 5 system. The expression levels of genes were measured using the comparative Ct method. Expression values were normalized to mouse glyceraldehyde-3-phosphate dehydrogenase (*Gapdh*) expression. The primers were designed with the help of Primer Premier 6 and are the same as those used in our previous reports [28]. The primer sequences are shown in Table 1.

### 4.15. Statistical Analysis

A Student’s *t*-test, one-way ANOVA test and Wilcox rank sum test were used as indicated in the figure legends. *p* < 0.05 was considered statistically significant.

## Figures and Tables

**Table 1:** Primer pair sequences for osteogenic transcription factors.

Name	Forward 5′-3′	Reverse 5′-3′
*Runx family transcriptional factor 2 (Runx2)*	AGAGTCAGATTACAGATCCCAGG	TGGCTCTTCTTACTGAGAGAGG
*Osteocalcin (Ocn)*	CCACCCGGGAGCAGTGT	CTAAATAGTGATACCGTAGATGCGTTTG
*Osterix (Osx)*	TCTCAAGCACCAATGGACTCCT	GGGTAGTCATTTGCATAGCCAGA
*Glyceraldehyde-3-phosphate dehydrogenase* *(Gapdh)*	CATGGCCTTCCGTGTTCCTA	CCTGCTTCACCACCTTCTTGAT

## Data Availability

Data sharing not applicable.

## Supplementary Materials

The supporting information can be downloaded at: https://www.mdpi.com/article/10.3390/ijms24097707/s1.
