# Supplementary material for: Synergistic Effect of QNZ, an Inhibitor of NF-κB Signaling, and Bone Morphogenetic Protein 2 on Osteogenic Differentiation in Mesenchymal Stem Cells through Fibroblast-Induced Yes-Associated Protein Activation
Source: Int J Mol Sci. 2023 Apr 22;24(9):7707. doi: 10.3390/ijms24097707 (PMC10178388; doi:10.3390/ijms24097707)
Supplement: Supplementary file 1 [file ijms-24-07707-s001.zip › ijms-2308448-supplementary.pdf]

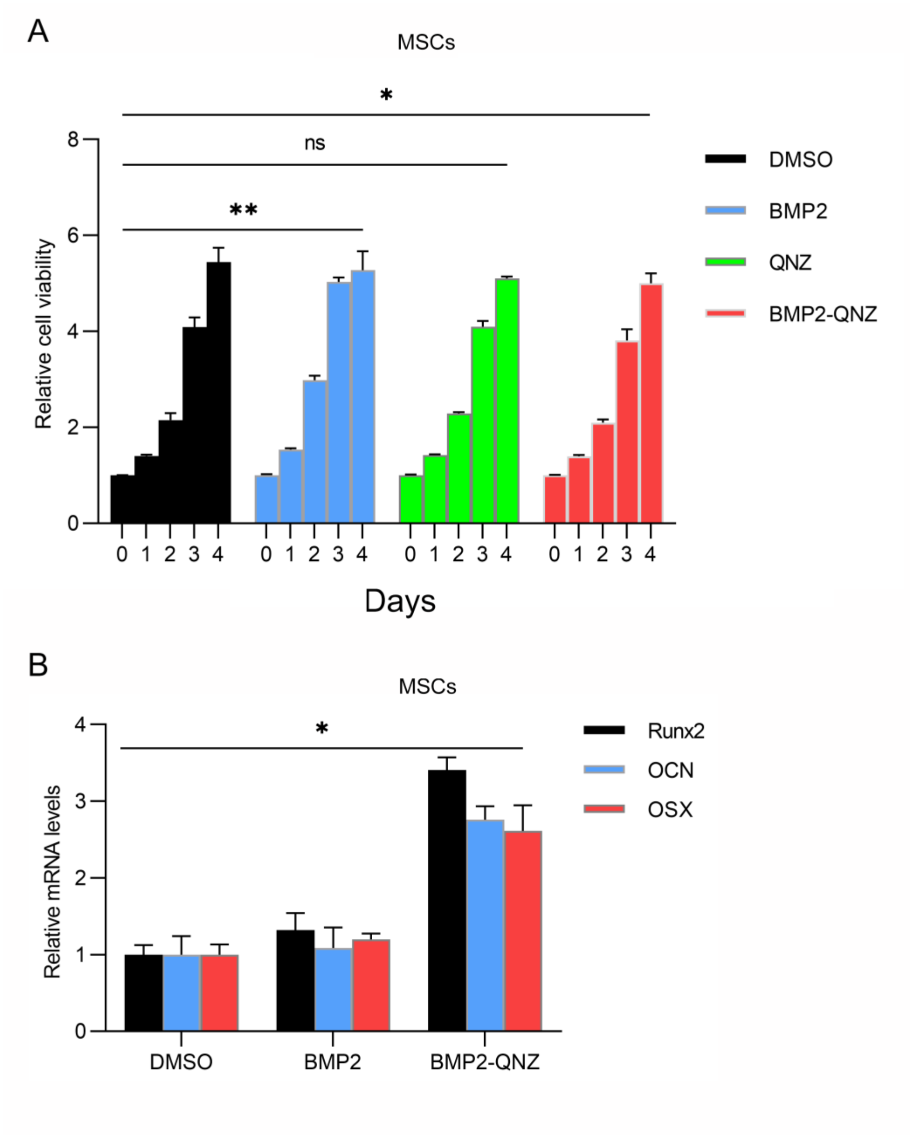

Figure S1. QNZ reversing the inhibition of fibroblasts in the BMP2-mediated osteoblast differentiation of MSCs.

(A) About 3,000 MSCs cultured in each well of 96 well plates with a control vehicle, 200 ng/mL of BMP2 or 10 μM QNZ, for the indicated time. Relative cell viability (OD values at 450 nm of each group normalized to the vehicle treated group) was measured with a cell-counting kit. At least three experiments were conducted and analyzed (n=3).*: *P<0.05*; **: *P<0.01*

(B) Relative mRNA levels of *Runx2*, *Ocn* and *Osx* after MSCs were directly co-cultured with MEFs and treated with control vehicle, 200 ng/mL of BMP2 or 10 μM QNZ. *: *P<0.05*

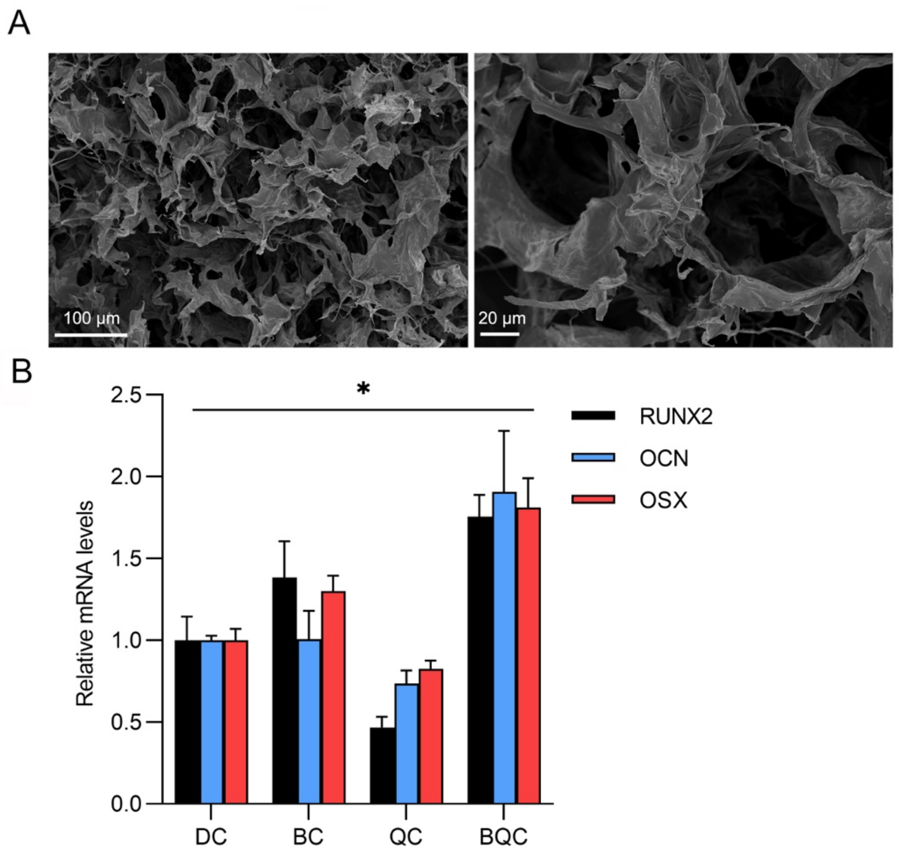

Figure S2. BMP2/QNZ/Collagen I biomaterials enhanced osteoblast differentiation of MSCs when MSCs co-cultured with MEFs.

(A) SEM observation illustrating that BMP2/QNZ/Collagen I biomaterials had several pores.

(B) Relative mRNA levels of *Runx2*, *Ocn* and *Osx* after MSCs were directly co-cultured with MEFs and treated with DC, BC, QC and BQC. *: $P<0.05$
